# Cloning and characterization of *Escherichia coli *DUF299: a bifunctional ADP-dependent kinase - P_i_-dependent pyrophosphorylase from bacteria

**DOI:** 10.1186/1471-2091-11-1

**Published:** 2010-01-03

**Authors:** Jim N Burnell

**Affiliations:** 1 Department of Biochemistry and Molecular Biology, James Cook University, Townsville, Queensland 4811, Australia

## Abstract

**Background:**

Phosphoenolpyruvate synthetase (PEPS; EC 2.7.9.2) catalyzes the synthesis of phosphoenolpyruvate from pyruvate in *Escherichia coli *when cells are grown on a three carbon source. It also catalyses the anabolic conversion of pyruvate to phosphoenolpyruvate in gluconeogenesis. A bioinformatics search conducted following the successful cloning and expression of maize leaf pyruvate, orthophosphate dikinase regulatory protein (PDRP) revealed the presence of PDRP homologs in more than 300 bacterial species; the PDRP homolog was identified as DUF299.

**Results:**

This paper describes the cloning and expression of both PEPS and DUF299 from *E. coli *and establishes that *E. coli *DUF299 catalyzes both the ADP-dependent inactivation and the P_i_-dependent activation of PEPS.

**Conclusion:**

This paper represents the first report of a bifunctional regulatory enzyme catalysing an ADP-dependent phosphorylation and a P_i_-dependent pyrophosphorylation reaction in bacteria.

## Background

In C_4 _plants, pyruvate, orthophosphate dikinase (PPDK; EC 2.7.9.1) catalyses the conversion of pyruvate to phosphoenolpyruvate (PEP) in what is generally recognized as the rate-limiting step in C_4 _photosynthesis [1] according to reaction 1.

Reaction 1....   Pyruvate + ATP + P_i_ ↔ PEP + AMP + PP_i_

In turn, PPDK activity is regulated by light via a rather unique phosphorylation/dephosphorylation mechanism. The regulatory mechanism involved differs from other phosphorylation/dephosphorylation mechanisms in a number of ways. Firstly, the regulatory mechanism uses ADP rather than ATP as the donor of a phosphate group. Secondly, the substrate (PPDK) for inactivation is a catalytically-phosphorylated form of the enzyme substrate. Thirdly, the activation reaction involves a phosphate-dependent phosphorolytic removal of the regulatory phosphate group rather than a simple phosphatase-catalysed dephosphorylation reaction. And fourthly, both the inactivation and activation activities are catalyzed by a single enzyme (see [2] for a review). The PDRP from maize [3] and Arabidopsis [4] have recently been cloned and expressed and their homology to the DUF299 gene family recognised.

A phylogenetic analysis of the DUF299 amino acid sequences available in GenBank segregated the DUF299 proteins into two major clades representing those bacterial species that possess PPDK and those that possess phosphoenolpyruvate synthetase (PEPS; EC 2.7.9.2) (see Results and Discussion below). PEPS is an enzyme found in many bacteria and catalyzes the phosphorylation of pyruvate to PEP according to reaction 2.

Reaction 2......   Pyr + ATP ↔ PEP + AMP + P_i_

Although there are varying degrees of homology between PPDKs and PEPSs the two types of enzyme can be discriminated by signature sequences identified by Tjaden et al [5]. An examination of the location of the *duf299 *gene in the genome of a large number of bacteria revealed that the gene is often, but not always, located close to either the *peps *or the *ppdk *gene. It is also interesting to note that although many members of the Archaea possess either the *ppdk *or *peps *gene they do not contain the *duf299 *gene.

PEP synthetase is present in many bacteria and has an important role in gluconeogenesis when bacteria are grown on small carbon substrates [6]. *E. coli *mutant studies demonstrated that PEPS-deficient mutants were unable to grow on pyruvate, lactate or alanine [7]. Cooper and Kornberg [8] also suggested that the reaction catalysed by PEPS involved the transfer of a phosphoryl-group from ATP to the enzyme and a phosphorylated form of the enzyme was isolated [8]. The formation of an EP form of the enzyme either in the presence of ATP or PEP was subsequently reported [9] and a histidine residue identified as the site of phosphorylation [10].

The successful expression of the maize PDRP (DUF299) and the close similarity of the amino acid sequence of bacterial DUF299 prompted an investigation of the role of the DUF299 from *E. coli*. This paper reports experiments performed with *E. coli *PEPS and DUF299 that clearly demonstrate that *E. coli *PEPS is controlled by a phosphorylation/dephosphorylation mechanism similar to that found in plants. And finally, given its function, it is proposed that this protein be given the common name of the PEP synthetase regulatory protein and the abbreviation PSRP be used to discriminate it from PDRP, DUF299 proteins that may catalyse the regulation of PPDK.

## Results and discussion

### Phylogenetic analysis of DUF299 amino acid sequences

**Figure 1:**
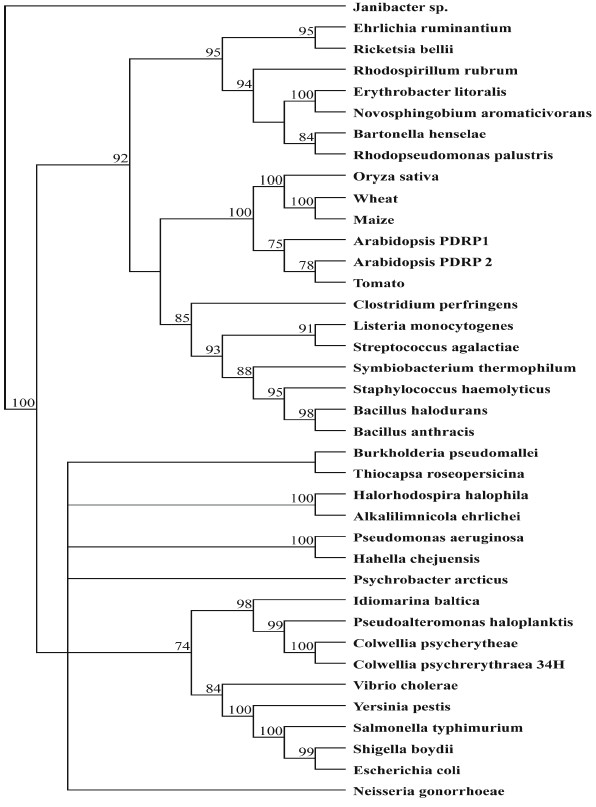
**Phylogenetic relationships of bacterial and plant DUF299 proteins**. The tree was constructed using a neighbour joining Poisson-corrected distance matrix method, with gaps distributed proportionally (MacVector, Accelrys).

A phylogenetic analysis of DUF299 amino acid sequences from a range of plant and bacterial species revealed divergence in amino acid sequences that segregated into two major clades (Figure 1). Further examination of the genomes of the species revealed that the DUF299-containing species segregated according to whether they possessed either PPDK (the upper half of the tree) or PEPS (the lower half of the tree); the identity of PPDK and PEPS was based on previously identified signature sequences [5].

### Cloning and expression of PEPS and PSRP

The DNA sequences of the PEPS and PSRP inserts in pROEXa were confirmed by DNA sequencing. Modifications to standard expression and extraction protocols were required to maximize the production of soluble, stable forms of both enzymes. PEPS expression was optimised by initially growing cells at 37°C for 3 h and pre-cooling cultures to 25°C prior to isopropyl thiogalactoside (IPTG) induction of protein synthesis. In addition, dithiothreitol (DTT), to a final concentration of 10 mM, and glycerol, to a final concentration of 20% (v/v), was required to be added to the purified protein to retain active, soluble protein. The purified protein was stable for several days at room temperature or for more than 6 months at -80°C. Cold stored enzyme needed to be reactivated by incubation at room temperature for at least 20 min.

For PSRP, cultures had to be cooled to 18°C prior to induction of protein synthesis at 18°C, and glycerol had to be included in all solutions used during the purification of the protein, to maintain the enzyme in a soluble form. The purified protein was stable for at least a week when stored on ice.

The MW of the PSRP subunits were about 25 kDa as determined by SDS-PAGE. The native size of the *E. coli *PSRP as determined by Sephacryl S200 gel filtration indicated the native form of the protein is a tetramer eluting from the column between lactate dehydrogenase (MW 140 kDa) and malate dehydrogenase (MW 67 kDa). It is not known whether the quaternary structure of the *E. coli *PSRP changes with pH; the maize PDRP is a dimer at pH 7.5 and a tetramer at pH 8.3 [11]. There was no evidence to suggest that the N-terminal His_6_-tag of pROEX, which included the amino acid sequence MSYYHHHHHHDYDIPTTENLYFQGA, affected PSRP activity in any way.

### Inactivation and activation activities of the expressed *E. coli *PSRP

The purified PSRP catalysed both the ADP/ATP-dependent inactivation (Table 1) and the P_i_-dependent activation of purified *E. coli *PEPS (Table 2). In addition, the expressed PSRP catalysed in-assay activation. In-assay activation occurs when inactivated PEPS in aliquots taken from a separate inactivation reaction activates in the assay used to measure PEPS activity. Addition of P_i _to reaction mixtures used to measure PEPS activity in aliquots taken from inactivation reactions resulted in an increasing rate of PEPS activity; in the absence of added P_i _PEPS activity remained linear. Therefore it was significantly easier to measure PEPS inactivation compared to measuring maize PPDK regulation due to the fact that there was no in-assay activation if P_i _was omitted from reactions measuring PEPS activity. Therefore, in contrast to measuring maize PDRP activity, there was no need to add Blue Dextran or Cibacron blue to inhibit in-assay activation when inactivation activities of *E. coli *PEPS were measured.

**Table 1:** ADP/ATP-dependent inactivation of bacterially-expressed *E. coli *PEPS by bacterially-expressed *E. coli *PSRP.

Reaction mixtures	PEPS activity at zero time (%)	PEPS activity after 5 min (%)	PEPS activity after 10 min (%)
Complete	100	16	4

Minus ADP/ATP	100	98	103

Minus PSRP	100	97	98

Minus PEPS	0	0	0

Complete plus 5 mM pyruvate	100	95	94

PEPS inactivation was measured as described in Materials and methods. The experiment was repeated five times and results presented in the table are from one experiment that was representative of all five experiments.

**Table 2:** P_i_-dependent activation of inactivated *E. coli *PEPS

Reaction mixtures	PEPS activity at zero time (%)	PEPS activity after 5 min (%)	PEPS activity after 10 min (%)
Complete (plus 1 mM P_i_)	6	83	92

Minus 1 mM Pi	6	11	15

Plus Pi and 2 mM ADP	5	13	17

Plus Pi and 2 mM AMP	6	18	27

The experiment was repeated five times; the results of one experiment, representative of all five experiments, are presented in the table.

**Figure 2:**
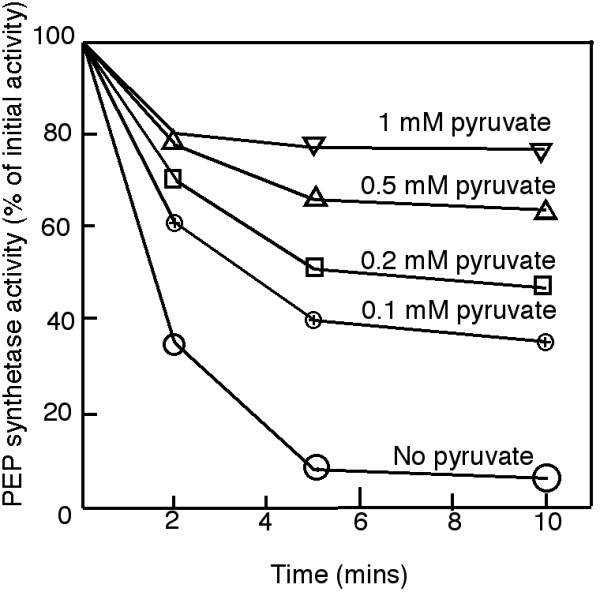
**Effect of pyruvate on the ADP-dependent inactivation of *E. coli *PEP synthetase**. Purified PEPS and DUF299 were incubated in the presence of 0.65 units of PEPS, 0.5 mg DUF299, 25 mM Hepes-KOH, 5 mM MgCl_2_, 5 mM DTT at pH 8.0. Inactivation was initiated by adding ADP and ATP to a final concentration of 2 and 0.1 mM, respectively. Pyruvate was added at the concentrations indicated. Experiments were conducted at least five times and the results presented in this figure are representative of the results obtained.

Addition of pyruvate to inactivation reactions inhibited ADP/ATP-dependent inactivation of *E. coli *PEPS (Figure 2). This result is similar to the pyruvate-dependent inhibition of PDRP-catalysed regulation of maize leaf PPDK [12] and indicated that

**Figure 3:**
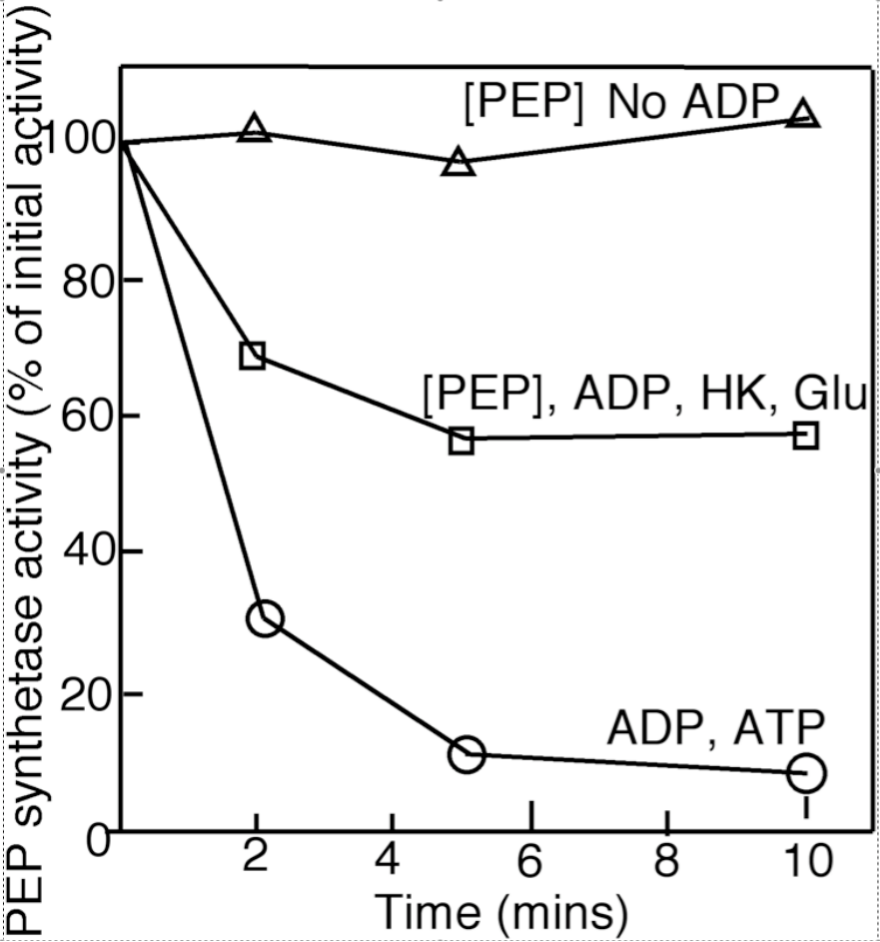
**Effect of PEP pre-treatment on the ADP-dependent inactivation of PEPS**. Purified PEPS was incubated with PEP (as shown by the square brackets) and the ATP removed by Sephadex G25 gel chromatography. The PEPS was then incubated in the presence of 25 mM Hepes-KOH, 5 mM MgCl_2_, 5 mM DTT at pH 8.0. Additions as indicated were 2 mM ADP, 2 mM glucose (Glu), 5 units of hexokinase (HK) and 0.1 mM ATP. Experiments were conducted at least three times and the results presented in this figure are representative of the results obtained.

*E. coli *PEPS had to be catalytically-phosphorylated prior to being inactivated. This was confirmed by pre-incubating PEPS with PEP, removing the PEP by Sephadex G25 column chromatography and subjecting the catalytically-phosphorylated PEPS to ADP-dependent inactivation in the presence of hexokinase and glucose; glucose and hexokinase were added to remove any ATP that may have been formed from ADP in the presence of contaminating adenylate kinase (Figure 3). Only about 40% of the PEPS was inactivated which suggested that less than 50% of the PEPS was catalytically phosphorylated during pre-incubation with PEP. This result was investigated further and it was shown that a small amount of P_i _and AMP present in the assay (presumably from the degradation of ADP) was responsible for the dephosphorylation of the catalytic histidine residue of PEPS.

Compared to the regulation of maize leaf PPDK by PDRP, the inactivation of *E. coli *PEPS by *E. coli *PSRP was considerably more sensitive to inhibition by pyruvate (see Figure 3), being almost five times more sensitive than maize PDRP (see [13]).

Inclusion of ADP (1 mM final concentration) in activation assays demonstrated that ADP inhibited P_i_-dependent activation (results not shown). This result is consistent with the ADP-dependent inhibition of P_i_-dependent activation observed with maize PDRP [2].

In comparing the *E. coli *PSRP to other DUF299-family members, *E. coli *PSRP resembles maize PDRP in that it catalyses both the ADP-dependent inactivation and the P_i_-dependent activation of its substrate enzyme. This is in contrast to one of the two Arabidopsis PDRP isozymes that have been reported [4], one of which catalyses both the inactivation and activation reactions while the second isozyme catalyzes only the inactivation reaction.

### Substrate specificity of *E. coli *PSRP and maize leaf PDRP

Experiments were conducted in which *E. coli *PSRP was replaced with bacterially-expressed maize PDRP [3]; no ADP/ATP-dependent inactivation of *E. coli *PEPS was detected (results not shown). Furthermore, no ADP/ATP-dependent inactivation was detected in reciprocal experiments in which *E. coli *PEPS was replaced with maize PPDK.

The fact that *E. coli *PEPS activity is regulated by PSRP may be important in light of experiments in which potato plants were transformed with an active *E. coli *PEPS in an attempt to increase the photosynthetic CO_2 _assimilation rates [14]; no increases in photosynthetic CO_2 _assimilation rates were detected. *E. coli *PEPS was introduced into potato (a C_3 _plant) in attempts to introduce an operating C_4 _photosynthetic pathway under the belief that the bacterial enzyme was not regulated [14].

**Figure 4:**
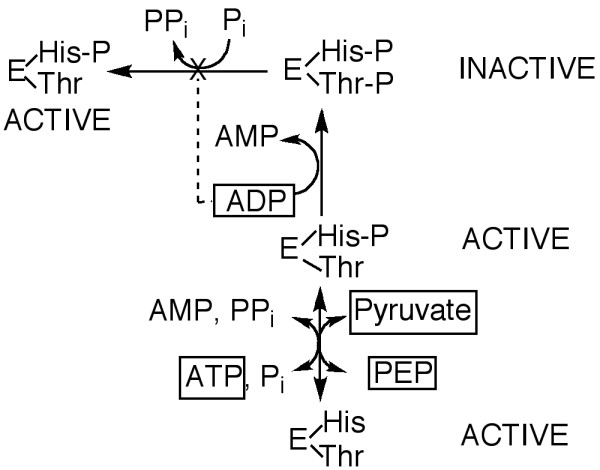
**Scheme for the regulation of *E. coli *PEPS by ADP, pyruvate and ATP**. The phosphorylation status of the catalytically-important histidine residue (His) and the regulatory threonine (Thr) residue of PEPS is shown and the location of the ATP, ADP and pyruvate is highlighted in boxes.

The regulation of *E. coli *PEPS by AMP, ADP, oxaloacetate, α-ketoglutarate, malate, ADP-glucose and 3-phosphoglyceraldehyde has been reported [15]. The results presented in this paper provide evidence for the existence of another level of regulation of PEPS activity. The PSRP-dependent regulatory mechanism may be critical in controlling the metabolic direction of pyruvate in the cell, either towards the oxidative catabolism of pyruvate via the pyruvate dehydrogenase complex to produce more ATP, or the anabolic conversion of pyruvate to PEP and glucose via an active PEPS. The results in this paper indicate that the PSRP-dependent regulation of PEPS in *E. coli *is affected by the concentration of three major compounds (see Figure 4); ADP, ATP and pyruvate. This regulation is similar to the PDRP-dependent regulation of PPDK in maize. In maize, ADP is both a substrate for the inactivation of PPDK and an inhibitor of the P_i_- dependent activation of inactivated PPDK. In contrast, ATP is not only a substrate for PPDK but, together with pyruvate, controls the rates of ADP-dependent inactivation of PEPS by controlling the phosphorylation status of the catalytic histidine residue; ADP-dependent inactivation of PEPS requires the catalytic histidine residue to be phosphorylated. The pyruvate-dependent inhibition of ADP-dependent inactivation of PEPS is consistent with *E. coli *PEPS needing to be catalytically phosphorylated prior to inactivation. Therefore when *E. coli *is grown on pyruvate or lactate as a sole carbon source, the metabolic fate of pyruvate will be controlled by the relative activities of pyruvate dehydrogenase and PEPS. Under elevated intracellular ADP concentrations ADP would not only inhibit PEPS activity but also inactivate PEPS while the pyruvate dehydrogenase complex would be active. In contrast, at low ADP concentrations, which would also indicate high ATP concentrations, PEPS would be activated and the E1 component of the PDH complex inhibited which would favour the anabolic conversion of pyruvate to PEP; PEP would be available for the shikimic acid pathway in addition to conversion to glucose.

At present, the phosphorylation status of the catalytic histidine residue of inactivated PEPS as a substrate for P_i_-dependent activation is not clear and is currently being investigated. In maize, the form of inactivated PPDK that is not phosphorylated on the catalytic histidine residue is the preferred substrate for Pi-dependent activation [12].

The synthesis of PEP via PEPS is important in the biosynthesis of many commercially important chemicals; PEP is a precursor for shikimic acid synthesis that, in turn, is the starting material for the production of a wide range of products including Tamiflu, an orally effective anti-influenza agent (see [16]). *E. coli*, over-expressing PEPS, are widely used in the biosynthesis of shikimic acid as is over-expression of 3-deoxy-D-arabino-heptulosonic acid 7-phosphate (DAHP) synthase whose use can be limited by feedback inhibition. Over-expression of feedback insensitive DAHP synthase isozymes have been used in microbial syntheses of commercially important products such as aromatic amino acids, phenylalanine and tryptophan [17]. The discovery of the existence of a phosphorylation/dephosphorylation mechanism that regulates PEPS activity may have to be taken into account if PEPS and PSRP are expressed in cells used for the synthesis of compounds dependent on the shikimic acid pathway. And in much the same way that feedback inhibition insensitive DAHP synthase isozymes are used in microbial based syntheses, PEPS may need to be altered to render the enzyme insensitive to PSRP-dependent regulation and maintain it in its active form. The effect of altering the regulatory threonine residue close to the catalytic histidine residue in PEPS is currently under study.

The wide distribution of DUF299 in bacteria, the proximity of the *duf299 *gene to *ppdk *and *pps *genes in bacterial genomes, the expression of *E. coli *PEPS and DUF299 and the demonstration that *E. coli *PEPS is subject to both ADP-dependent inactivation and P_i_-dependent activation, indicate that the function of the gene family identified as DUF299 is to control either PEPS or PPDK activities. The DUF299 from *Streptococcus agalactiae*, a bacterial species possessing both PPDK and DUF299, is currently being investigated.

## Conclusions

A phylogenetic analysis of the *duf299 *gene, present in most bacterial species, segregates bacterial species into two major clades; those possessing PPDK and those possessing PEPS. Expression of the *duf299 *gene from *E. coli *resulted in the synthesis of an enzyme that catalysed both ADP-dependent inactivation of *E. coli *PEPS and P_i_-dependent activation of inactive *E. coli *PEPS. Experiments revealed that the PSRP from *E. coli *was very similar to the PDRP from maize in that the enzyme substrate of the regulatory protein had to be catalytically-phosphorylated before it could act as a substrate for ADP-dependent phosphorylation. In addition, ADP inhibited the Pi-dependent activation activity of DUF299. The *E. coli *PSRP was shown to be active as a tetramer but was not capable of catalysing either the ADP-dependent inactivation or the Pi-dependent activation of maize leaf PPDK.

Since PSRP and PDRP are expressed in a large number of bacteria many of which are pathogenic, and since the enzymes are rather unique in the types of reactions they catalyse, it may be possible to identify compounds that may selectively inhibit the enzymes inhibiting the synthesis of important biochemical intermediates and ultimately inhibiting the growth of the bacteria.

## Methods

### Phylogenetic analysis of DUF299 amino acid sequences

DUF299 amino acid sequences were downloaded from either GenBank or Integrated Microbial Genomes and analysed using a neighbour joining Poisson-corrected distance matrix method, with gaps distributed proportionally (MacVector, Accelrys).

### Cloning of *E. coli *PEPS and PSRP

*E. coli *genomic DNA was amplified by PCR using EC primers 1 and 2 (5'-GGATTGTTCCATGGCCAACAATGG-3', 5'-GCCGCATCATTCATTATCGC-3', respectively) and the 2.6 kbp product ligated into pGEM-T. DNA was amplified in NM522 cells, plasmid DNA isolated and the DNA sequence determined (Macrogen, Korea). Following confirmation of the DNA sequence, plasmid DNA was digested with *Nco*I and *Spe*I and two DNA bands isolated (a 5'-end 700 bp *Nco*I-*Nco*I band and an 1800 bp NcoI-*Spe*I band. The 1800 bp *Nco*I-*Spe*I band was ligated into *Nco*I-*Spe*I digested pROExa and the incorporation of the insert confirmed by DNA sequencing. The pROExa containing the insert was digested with *Nco*I and ligated with the 700 bp *Nco*I-*Nco*I fragment isolated from the pGEMT-PEPS clone and the correct orientation of the *Nco*I-*Nco*I insert confirmed by DNA sequencing of the resulting plasmid.

For PSRP, *E. coli *genomic DNA was amplified using PCR primers 3 and 4 (5'-GGGAAGAATTCATGGATAATGCTGTTGAT-3' and 5'-TGATTTCAAGTGCGAGGTGTGTC-3', respectively). The PCR product was isolated using a Qiagen Plasmid Miniprep Kit and the eluted DNA digested with *Eco*RI and *Spe*I. The *Eco*RI-*Spe*I fragment was ligated into *Eco*RI-*Spe*I digested pROEXa overnight, NM522 cells transformed and plasmid DNA isolated from cultured colonies. The DNA sequence of pROEXa DNA was analysed (Macrogen, Korea).

### Cloning of maize PPDK and PDRP

Maize leaf PPDK and PDRP were expressed in *E. coli *and purified as previously described ([3] and [18], respectively).

### Protein expression and enzyme purification

Cells containing the pROEXa-PEPS plasmid were cultured overnight in 5 mL LBA broth (LB plus 75 μg.mL^-1 ^ampicillin) and used to inoculate 500 mL LBA in 2 L baffled flasks. Cultures were shaken (200 rpm) at 37°C for 3 hr, the cultures cooled to 25°C prior to the addition of IPTG to a final concentration of 1 mM and shaken for 24 h. Cells were harvested by centrifugation (4, 000 × g for 10 mins), resuspended in PEPS Column buffer (25 mM Tris-HCl, pH 7.0, 200 mM NaCl, 5 mM MgCl_2 _and 10 mM β-mercaptoethanol), pelleted by centrifugation, resuspended in 15 mL of column buffer and frozen at -80°C. Following thawing, cells were broken by sonication on ice and the cell debris removed by centrifugation (40, 000 × g for 20 min). The supernatant was filtered (22 μm filter) and loaded onto a 5 mL Nickel-NTA column equilibrated with buffer at a flow rate of 1.0 mL.min^-1^. The column was washed with buffer until the OD_280 nm _decreased below 0.05, with buffer containing 20 mM imidazole until the A_280 nm _decreased below 0.05 and protein eluted with column buffer containing 200 mM imidazole. DTT was added to each eluted fraction (2.5 mL) to a final concentration of 10 mM and column eluate stored at -20°C or -80°C until required.

Cells containing the pROEXa-PSRP plasmid were cultured overnight in 5 mL LBA and used to inoculate 500 mL LBA in 2 L baffled flasks. Cultures were grown for 3 h at 37°C with shaking (200 rpm), the cultures cooled to 18°C prior to the addition of IPTG to a final concentration of 1 mM. Cultures were shaken at 18°C for 4 days, cells harvested by centrifugation and resuspended in PSRP Column buffer containing 25 mM Hepes-KOH, 20% v/v glycerol, 300 mM NaCl, 5 mM MgCl_2 _and 10 mM β-mercaptoethanol. Cells were disrupted using a French Press (pre-cooled to 2°C) and cell debris removed by centrifugation at 40, 000 × g for 30 mins at 2°C. Expressed PSRP was purified by nickel affinity chromatography as described for the purification of PEPS.

### Assay of enzyme activity

PEPS was assayed spectrophotometrically in a coupled enzyme assay in which the pyruvate-dependent production of PEP was linked to NADH oxidation via PEP carboxylase and malate dehydrogenase. All assays were conducted at 25°C in a Beckman DU650 spectrophotometer. Reactions mixtures contained 25 mM Tris-HCl, pH 8.0, 8 mM MgCl_2_, 10 mM DTT, 10 mM NaHCO_3_, 2 mM pyruvate, 1 mM glucose-6-phosphate, 1 mM ATP, and 2 mM NADH, 2 units of maize leaf PEPC and 2 units of malate dehydrogenase. Reactions were initiated by the addition of enzyme (PEPS or an aliquot of a PSRP reaction assay).

Inactivation reactions contained between 0.5 to 1 unit of PEPS, 25 mM Hepes-KOH, pH 8.0, 5 mM MgCl_2_, 5 mM DTT, 0.1 mM ATP, 2 mM ADP and a variable amount of PSRP in a total volume of 0.2 mL. Inactivation reactions were initiated by the addition of ADP/ATP and 20 μL aliquots removed at different time intervals and the PEPS activity measured as described above.

To measure PEPS activation, PEPS was first inactivated by incubation of PEPS with purified PSRP, ADP and ATP as described for an inactivation reaction for 20 minutes in which time >95% of the PEPS was inactivated. The entire inactivation reaction was desalted on a Sephadex G25 column (0.5 mm × 10 mm) equilibrated with column buffer, and 250 μL of column eluate collected after the void volume was discarded. Aliquots (50 μL) of Sephadex G25 column eluate were added to an equal volume of column buffer and activation initiated by the addition of P_i _(to a final concentration of 1 mM). Aliquots (20 μL) were removed and assayed for PEPS activity.

### Isolation of inactivated PEPS

A scaled up inactivation assay was run in which 0.2 mL of PEPS (15 units.mL^-1^) was inactivated in the presence of 0.2 mL purified *E. coli *PSRP and 20 mM ADP/1 mM ATP in a total volume of 0.5 mL and PEPS inactivated for 30 min. Less that 2.5% of the initial activity remained after the 30 min incubation. The entire inactivation reaction mixture was loaded onto a 5 mL column of Agarose-Blue dextran (Sigma-Aldrich) and the column washed with PEPS Column buffer. The PSRP bound to the column while the PEPS passed through the column and was separated from the ADP/ATP by gel filtration. The protein concentration of eluate was monitored at 280 nm and the protein peak collected and used as the supply of inactive PEPS in activation assays. The successful removal of PSRP was confirmed by adding a 50 μL aliquot of the peak protein fraction to a 1 mL PEPS assay mix and adding phosphate to a final concentration of 1 mM in the assay.

### Catalytic phosphorylation of PEPS by PEP

In experiments in which the PEPS was catalytically phosphorylated in the presence of PEP, 2 units of PEPS in PEPS Column buffer was incubated with 5 mM PEP for 20 mins at room temperature and the PEP removed from the PEPS by Sephadex G25 column chromatography.

### Determination of the native molecular weight of PSRP

A 0.5 mL sample of the fraction with the highest ADP-dependent PEPS inactivation activity eluted from the nickel-NTA column was loaded onto a column (1.5 cm × 60.0 cm) of Sephacryl-S200 equilibrated with PSRP Column buffer and 2.0 mL fractions collected at a flow rate of 0.5 mL.min^-1^. The column was calibrated with lactate dehydrogenase (MW 140, 000), malate dehydrogenase (MW 67, 000) and bovine erythrocyte carbonic anhydrase (MW 30, 000). Lactate dehydrogenase and malate dehydrogenase were assayed spectrophotometrically at 340 nm and carbonic anhydrase was measured by monitoring the change in pH [19].

The subunit MW of PSRP was determined by SDS-PAGE on a 10% polyacrylamide gel [20].
